# Evaluating the Genotoxic and Cytotoxic Effects of Thymidine Analogs, 5-Ethynyl-2′-Deoxyuridine and 5-Bromo-2′-Deoxyurdine to Mammalian Cells

**DOI:** 10.3390/ijms21186631

**Published:** 2020-09-10

**Authors:** Jeremy S. Haskins, Cathy Su, Junko Maeda, Kade D. Walsh, Alexis H. Haskins, Allison J. Allum, Coral E. Froning, Takamitsu A. Kato

**Affiliations:** Department of Environmental & Radiological Health Sciences, Colorado State University, Fort Collins, CO 80526, USA; haskins@rams.colostate.edu (J.S.H.); cathy50720@gmail.com (C.S.); junkorv0507@yahoo.co.jp (J.M.); kwalsh1@rams.colostate.edu (K.D.W.); 2013ahaskins@gmail.com (A.H.H.); allison10allum@gmail.com (A.J.A.); coral.froning@gmail.com (C.E.F.)

**Keywords:** EdU, BrdU, radiosensitizer, DNA repair

## Abstract

BrdU (bromodeoxyuridine) and EdU (ethynyldeoxyuridine) have been largely utilized as the means of monitoring DNA replication and cellular division. Although BrdU induces gene and chromosomal mutations and induces sensitization to photons, EdU‘s effects have not been extensively studied yet. Therefore, we investigated EdU’s potential cytotoxic and mutagenic effects and its related underlying mechanisms when administered to Chinese hamster ovary (CHO) wild type and DNA repair-deficient cells. EdU treatment displayed a higher cytotoxicity and genotoxicity than BrdU treatment. Cells with defective homologous recombination repair displayed a greater growth delay and severe inhibition of clonogenicity with EdU compared to wild type and other DNA repair-deficient cells. Inductions of sister chromatid exchange and hypoxanthine phosphorybosyl transferase (HPRT) mutation were observed in EdU-incorporated cells as well. Interestingly, on the other hand, EdU did not induce sensitization to photons to the same degree as BrdU. Our results demonstrate that elevated concentrations (similar to manufacturers suggested concentration; >5–10 μM) of EdU treatment were toxic to the cell cultures, particularly in cells with a defect in homologous recombination repair. Therefore, EdU should be administered with additional precautions.

## 1. Introduction

BrdU (Bromodeoxyuridine) has been utilized as a primary means of labeling actively dividing cells. Contrastingly, EdU (Ethynyldeoxyuridine) has developed popularity as a rapid and effective biochemical agent for detection of DNA synthesis [1,2,3]. EdU was first demonstrated to monitor and detect replicational activity in 2008 [2]. Of the potential advantages of EdU application, one can be explained and illustrated via Click reaction procedures: omission of the volatile DNA denaturation process that is required for BrdU detection by an antibody, results in an elevated infidelity of DNA recombination [2]. EdU has additional quantifiable and qualitative applications such as apoptosis detection [4], Raman spectroscopy [5,6], viral/bacterial inactivation [7], the DNA fiber assay [8], and sister chromatid exchange (SCE) assay [9,10,11]. Nonetheless, toxic effects of EdU are also reported throughout the literature [4,12,13,14].

Halogenated pyrimidines, including BrdU, CldU (Chlorodeoxyuridine), and IdU (Iododeoxyuridine), have similar atomic structures to that of thymidine [15,16]. These analogues are remarkably structurally similar to thymidine by the replacement of the 5-methyl group with a respective halogen [16]. Among them, the bromo-substituent of BrdU is closely identical to that of a methyl residue. However, halogenated pyrimidines including BrdU exhibit cytotoxic and mutagenic properties [17]. High concentrations of BrdU treatment increase SCE frequency [18] while influencing a variety of mutations [11,19]. Additionally, the application of BrdU has potent Poly (ADP-ribose) Polymerase (PARP) inhibitory properties [20]. BrdU, thymidine, and related 5′-halogenated analogs have altogether displayed an efficacious ability to affect PARP activity [20], which can be demonstrated by inducing genomic instability.

Moreover, cells incorporating halogenated pyrimidine are sensitized to photon exposure including light from fluorescent lamps [21], Ultraviolet (UV) light [22], and ionizing radiation [23]. Although the sensitization effect to BrdU is strongest among halogenated pyrimidines, the sensitization effect to gamma-rays is strongest in IdU [24]. Exposure to photons induces halogen radicals; these radicals induce strong biological effects such as DNA strand breaks [23]. Building evidence suggests that EdU retains inherent implications toward cell fidelity and viability such as increased apoptosis and DNA strand breaks in human glioblastoma cells, activating DNA damage response pathway in S. cerevisiae, and decreased transition (amount of) cells from G_2_-M phase [25,26,27]. Importantly, all of the cellular effects of EdU incorporation and phototoxicity have yet to be fully understood in vitro.

We provide evidence that EdU induces considerable genotoxic and cytotoxic stress to cells and produces a magnitude of effects in specific DNA repair-deficient cells. Interestingly, EdU did not affect cellular sensitivity to gamma-rays, UV-C, or light from a fluorescent lamp. Here, we investigate EdU as an alternative of BrdU. Discerning the potential, biochemical affects that EdU imposes on DNA replicative ability and cellular fidelity in a subset of DNA repair mutant cell lines.

## 2. Results

### 2.1. The In Vitro Effects of Nucleotide and Nucleoside Supplemented Medium on CHO Cells

**Figure 1:**
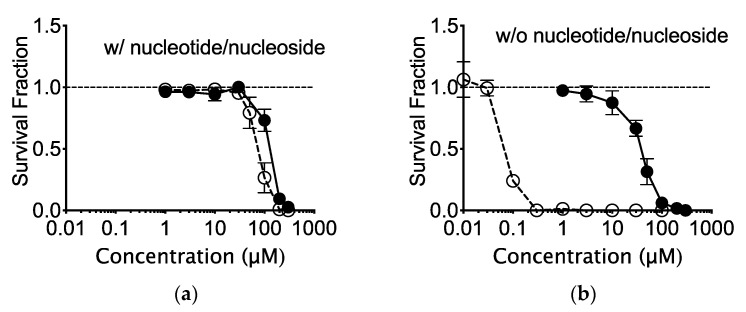
Cytotoxicity of bromodeoxyuridine (BrdU) and ethynyldeoxyuridine (EdU) to Chinese hamster ovary (CHO) cells. CHO cell survival against BrdU or EdU in the presence (**a**); or absence (**b**) of nucleotide/nucleoside supplement. Open circles indicate EdU and closed circles indicate BrdU. Error bars represent the standard error of the mean of three independent experiments.

AlphaMEM medium, supplemented with nucleoside and nucleotide, contains 10.0 mg/L thymidine (0.041 mM). In order to understand the cytotoxic effects of EdU on CHO cells, AlphaMEM medium (nucleotide/nucleoside deficient) was utilized during colony formation. BrdU cytotoxicity was enhanced in the absence of thymidine. There was nearly a 1000-fold decrease in survival fraction in cells treated with EdU and absence of nucleotide/nucleoside (Figure 1a,b).

### 2.2. Mutagenic Properties of EdU

**Figure 2:**
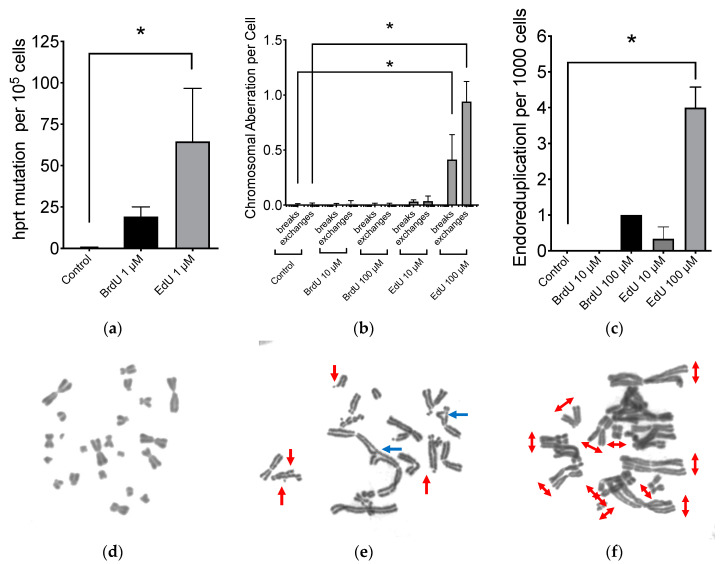
Genotoxicity of BrdU and EdU. (**a**) Hypoxanthine phosphorybosyl transferase (HPRT) mutation induction with BrdU and EdU; (**b**) chromosomal aberration formation in genomic instability 24 h after completing BrdU or EdU treatment; (**c**) endoreduplication formation after BrdU and EdU uptake. White bar indicates control. Black bar indicates BrdU treatment. Grey bar indicates EdU treatment. Error bars represent the standard error of the mean of three independent experiments. One-way ANOVA, Dunnett’s Multiple Comparison Test was performed to provide *p*-values. * indicates statistically significant differences (*p* < 0.05); (**d**) a representative image of CHO metaphase spread for control; (**e**) a representative image of genomic instability after EdU treatment. Red arrows indicate breaks and blue arrows indicate exchanges; (**f**) a representative image of EdU-induced endoreduplication. Red arrows indicate endoreduplicated chromosomes.

Mutagenicity of EdU was investigated with CHO HPRT mutation assay. Although high concentrations (10–100 μM of EdU) were too toxic to conduct this experiment, an increased HPRT mutation frequency was clearly demonstrated by DNA-incorporation of both 1 μM of BrdU and EdU without any cytotoxicity (Figure 2a). Background HPRT mutation frequency was 0.4 per 10^5^ cells. BrdU substitution increased HPRT mutation frequency to 19 per 10^5^ cells. EdU-induced HPRT mutation was approximately 65 per 10^5^ cells. Even at concentrations as low as 1 μM EdU, there was a recognizable increase in HPRT mutation. In the same respect, concentrations of 1 μM BrdU still demonstrated a noticeable induction of HPRT mutations.

Administration of 100 μM of BrdU or EdU treatment to CHO cells were carried out for 24 h and media was replenished with fresh media without BrdU or EdU for an additional 24 h (Figure 2b). Although this short-term treatment of BrdU or EdU did not cause any cytotoxicity, chromosomal aberration frequency was significantly higher in EdU-treated cells. On the other hand, BrdU-treated cells did not show statistically significant increases. Chromatid type aberrations including breaks and exchanges were observed with EdU treatment (Figure 2d). Additionally, endoreduplication formation was observed with EdU treatment (Figure 2c,e). BrdU treatment also increased endoreduplication in metaphase chromosomes. EdU induced approximately four times more endoreduplication compared to BrdU.

### 2.3. Effect to DNA Damage Responses

**Figure 3:**
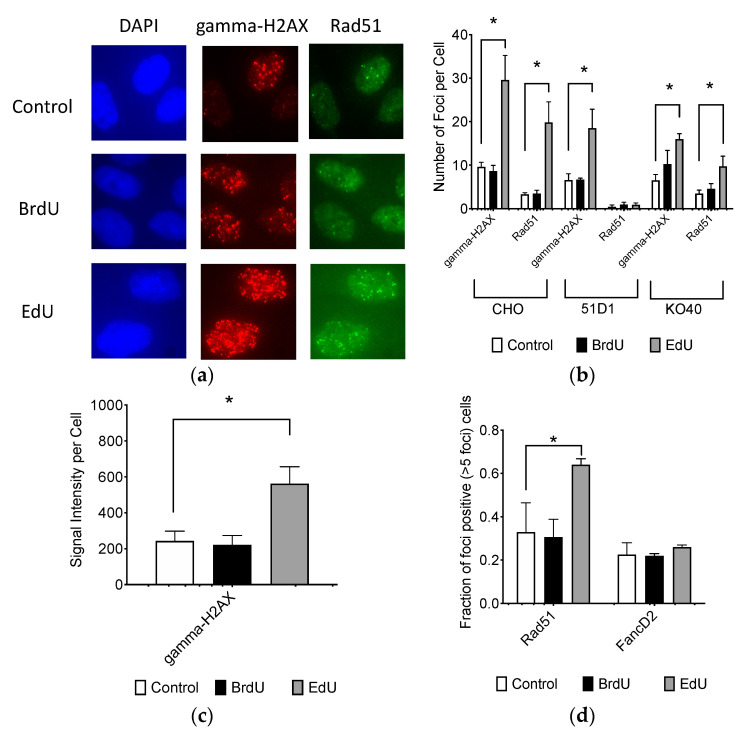
DNA damage and response after BrdU and EdU treatment. (**a**) Immunocytochemistry images after 10 μM BrdU or EdU treatment visualized with DAPI (blue), gamma-H2AX (red) and Rad51 (green) for CHO cells; (**b**) quantitative DNA damage response analysis of 10 μM BrdU or EdU treatment for CHO, 51D1, and KO40 cells; (**c**) signal intensity analysis of CHO cells; (**d**) populations of Rad51-positive (foci more than 5 per cell) cells after 10 μM BrdU or EdU treatment for CHO cells. White bar indicates control. Black bar indicates BrdU treatment. Grey bar indicates EdU treatment. Error bars represent the standard error of the mean of three independent experiments. One-way ANOVA, Dunnett’s Multiple Comparison Test was performed to provide *p*-values. * indicates statistically significant differences (*p* < 0.05).

CHO cells treated with BrdU or EdU were investigated for DNA damage responses including gamma-H2AX foci formation and Rad51 foci formation with fluorescent immunocytochemistry (Figure 3a). Although 10 μM of BrdU treatment for 24 h did not increase gamma-H2AX or Rad51 foci formation compared to the control, 10 μM of EdU significantly increased both gamma-H2AX and Rad51 foci numbers (Figure 3b). Results of manual foci analysis was confirmed with signal intensity analysis (Figure 3c). Rad51 foci were colocalized with gamma-H2AX foci in nuclei. Populations of Rad51 foci-positive cells (more than 5 foci per cell), also showed EdU-induced homologous recombination repair activity (Figure 3d). However, FancD2 foci-positive cells were not increased with EdU treatment. 51D1 cells formed minimal amounts of Rad51 foci for background and BrdU/EdU treatment. EdU induced gamma-H2AX foci for 51D1 and KO40. KO40 formed EdU-induced Rad51 foci. This suggests that EdU is implicated in an increased genotoxic response and activation of DNA repair machinery compared to BrdU. Homologous recombination repair with functional Rad51 alleviates DNA damage response induced by EdU.

### 2.4. Effect of EdU on SCE Formation

**Figure 4:**
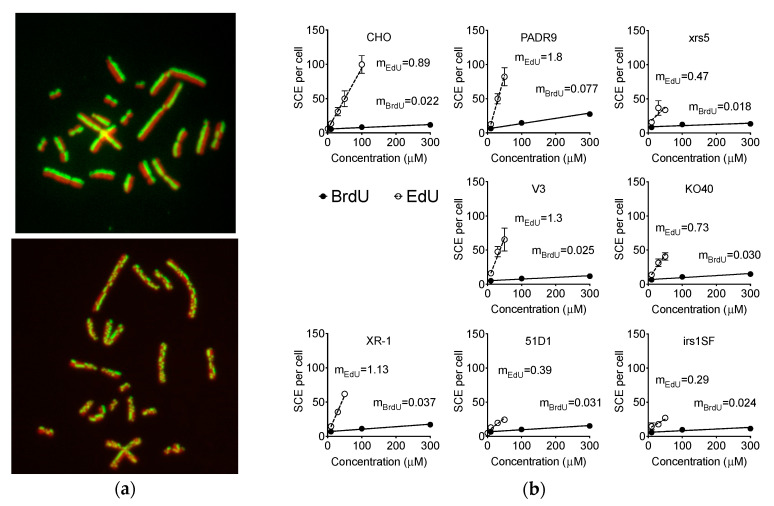
BrdU- and EdU-induced sister chromatid exchange (SCE) formation. (**a**) Representative images of control (upper panel) and 100 μM of EdU-induced (lower panel) SCE in CHO cells. Green signal indicates EdU and red signal indicates DNA staining with DAPI (pseudo-color); (**b**) SCE formation by BrdU and EdU in DNA repair-deficient cells. Closed circles indicate BrdU treatments and open circles indicate EdU treatments. Error bars represent the standard error of the mean of three independent experiments. m_BrdU_ and m_EdU_ indicate the values of slope in SCE per cell per μM of BrdU or EdU, respectively.

EdU-induced replication stress was confirmed by analysis of SCEs. The basal SCE frequency of 10 μM BrdU-treated CHO was 5.5 SCEs per cell. Notably, treatment of 1 μM and 10 μM EdU, CHO presented 5.5 and 12 SCEs per cell, respectively. Higher concentrations of BrdU (100 and 300 μM) induced 8 and 11 SCEs per cell, respectively. Surprisingly, CHO wild type cells displayed a much steeper EdU-dose-dependent increase in SCE frequency (m_EdU_ = 0.87 SCE per cell per μM of EdU) than that of BrdU (m_BrdU_ = 0.022 SCEs per cell per μM of BrdU). Moreover, 30 μM and 50 μM of EdU treatment induced 31 and 51 SCEs per cell, respectively. At 100 μM of EDU treatment, approximately 100 SCEs were observed per cell (Figure 4a,b).

DNA repair-deficient cells treated with BrdU displayed elevated SCE frequency at higher concentrations, especially in PARP-deficient PADR9 cells. Strikingly, these reported the highest SCE frequency (27 SCEs per cell) at 300 μM; being the highest EdU-dose-dependent increase compared to BrdU (m_EdU_ = 1.8 SCEs per cell per μM of EdU). Other DNA repair-deficient cells presented similar increases in SCE observed in the CHO wild type cells. With EdU treatment, all DNA repair-deficient cells induced SCE in a dose dependent manner (0–300 μM). The induction of SCE in KO40 and xrs5 cells was appropriately similar to the CHO wild type. NHEJ repair-deficient V3, XR1 and PARP-deficient PADR9 cells reported greater induction of SCE compared to CHO wild type. On the other hand, HR repair-deficient 51D1 and irs1SF presented lesser induction of SCE compared to wild type; m_EdU_ = 0.39 and 0.29, respectively.

### 2.5. Effect of DNA Repair for Cytotoxicity

**Figure 5:**
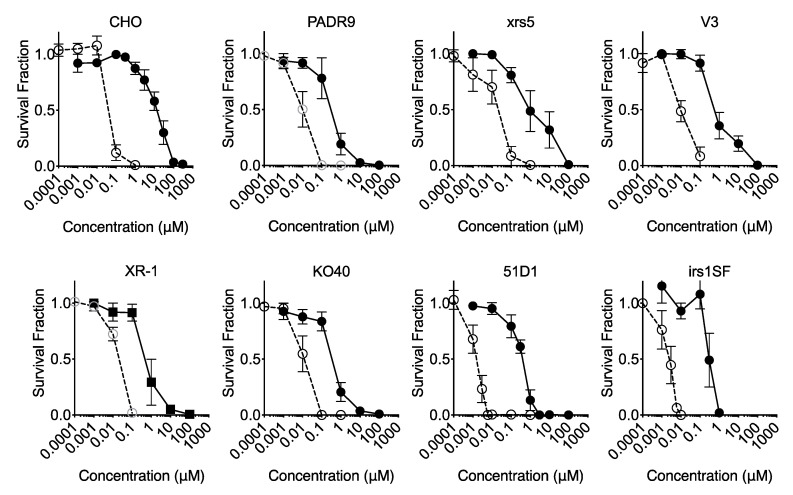
Cell survival curves against BrdU or EdU in DNA repair-deficient cells in the nucleotide and nucleoside free media. Closed circles indicate BrdU treatments and open circles indicate EdU treatments. Error bars represent the standard error of the mean of three independent experiments.

MEM media, voided of nucleotide/nucleoside, were used to observe maximum cytotoxicity of EdU and BrdU (Figure 5). CHO cells are viable and able to form colonies with IC_50_ of 15 μM BrdU. However, all DNA repair-deficient cells analyzed indicated similar values of IC_50_ among cell lines, with values approximately 0.30–0.63 μM (Table 1). Therefore, DNA repair deficiency increased sensitivity to BrdU by approximately 50-fold. For EdU treatment, CHO wild type cells showed the highest IC_50_ values of 88 nM. Among DNA repair-deficient cell lines, NHEJ repair-deficient V3, xrs5 and XR-1 cells showed IC_50_ values between 22 and 25 nM, respectively. Fanconi Anemia-deficient KO40 and PARP-deficient PADR9 cells showed IC_50_ = 11 and 10 nM. HR repair-deficient 51D1 and irs1SF cells showed severe replicative sensitivity to EdU; IC_50_ values were 2.1 and 2.7 nM, respectively. Therefore, DNA repair deficiency increased sensitivity to EdU from 4 to 40 fold. This suggests that all DNA repair mechanisms tested are approximately equally important for BrdU-induced stress and damages. On the other hand, homologous recombination repair is appreciably much more imperative than NHEJ repair for EdU-induced stress and damages. The ratio of IC_50_ revealed that loss of NHEJ, Fanconi Anemia, and PARP have stronger effects on cell death induced by BrdU compared to EdU.

### 2.6. Effect of EdU Continuous Treatment on Homologous Recombination-Deficient Cells

**Figure 6:**
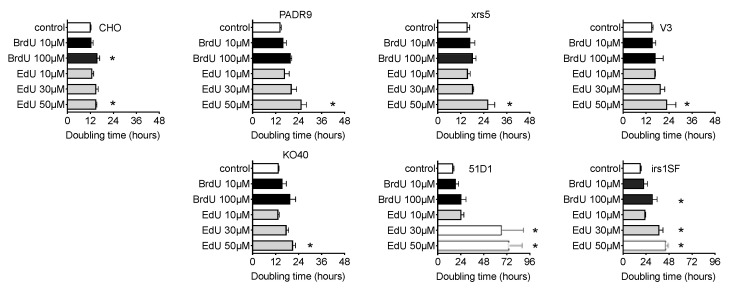
Cell doubling time and growth delay with BrdU and EdU treatment. CHO and DNA repair-deficient cells were grown with or without BrdU or EdU. Open bars indicate control. Black bars indicate BrdU treatment. Grey bars indicate EdU treatment. Error bars represent the standard error of the mean of three independent experiments. One-way ANOVA, Dunnett’s Multiple Comparison Test was performed to provide *p*-values. (*) denotes significance against control within each cell group (*p* < 0.05).

Both BrdU and EdU increased cell doubling time in a dose dependent manner (Figure 6). Administration of 10 μM BrdU or EdU, in the presence of nucleotide/nucleoside, did not alter cell doubling time significantly (*p* > 0.05). BrdU (100 μM) increased doubling time for all cell lines, but a statistically significant difference was observed in CHO wild type and irs1SF cells. Delay of cell doubling time was strongly observed in EdU-supplemented cells. An amount of, 50 μM of EdU showed severe growth delay in all cell lines, which were statistically significant differences compared to the basal level. Lastly, an EdU’s growth inhibitory effect was strongly observed in homologous recombination repair-deficient cell lines, 51D1 and irs1SF. Treatment of 50 μM of EdU, 51D1 and irs1SF presented doubling times of 75 and 45 h, respectively; their basal doubling times were 16 and 18 h, respectively.

### 2.7. Effect of EdU for SCE Formation and Chromosome Aberrations after the Second Cell Cycle

**Figure 7:**
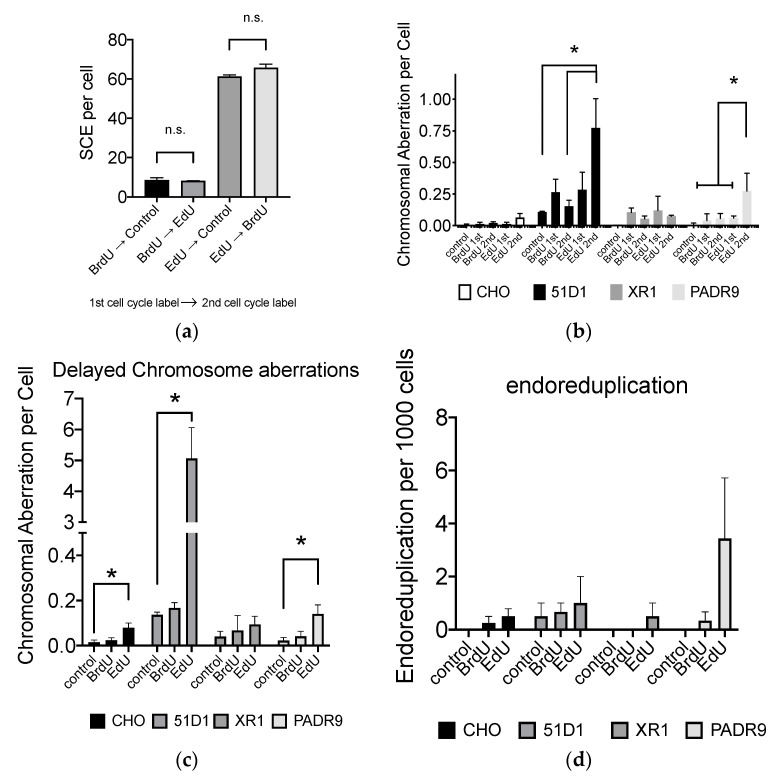
Delayed EdU effect for genomic damages. (**a**) The first cycle with BrdU or EdU determines SCE formation but not the second cycle. BrdU or EdU (50 μM) was incorporated; (**b**) EdU affects second post-treated metaphase and induces chromosomal aberrations. Chromosomal aberrations were compared with control, after one complete cycle of 10 μM BrdU or EdU treatment, and after two complete cycles of 10 μM BrdU or EdU treatment; (**c**) delayed chromosome aberration formation with DNA repair-deficient cells treated with 10 μM BrdU or EdU; (**d**) endoreduplication formation after 10 μM BrdU or EdU treatment. Error bars represent the standard error of the mean of three independent experiments. One-way ANOVA, Dunnett’s Multiple Comparison Test was performed to provide *p*-values. * indicates statistically significant differences. n.s. stands for not significant (*p* > 0.05).

In order to determine the timing of EdU-induced genomic damages, firstly, SCE formation by BrdU or EdU, CHO cells were treated with BrdU or EdU for the first cell cycle, then cultured with EdU or BrdU, respectively, or non nucleotide analog media (Figure 7a). The SCE formation was higher with EdU-treated cells in the first cell cycle than BrdU-treated cells. The BrdU or EdU incorporation in the second cell cycle did not show a significant increase in SCE formation. Therefore, SCE events occur when DNA replication utilizes the EdU- or BrdU-incorporated strands.

Secondly, chromosome aberrations were analyzed for the first post-labeling metaphases and the second post-labeling metaphases to investigate mechanisms involving EdU toxicity. Treatment of 10 μM BrdU and EdU did not produce a significant increase in chromosome aberrations for the first or second post-labeled metaphases in CHO wild type cells. However, increases in chromosome aberrations were observed in the second post-EdU-labeled metaphases for HR repair-deficient 51D1 cells (Figure 7b).

Further confirming delayed chromosome aberration formation in DNA repair mechanisms, CHO wild type, HR-deficient 51D1, NHEJ-deficient XR-1, and PARP-deficient PADR9 were investigated for delayed chromosome aberration formation as shown in Figure 2. DNA repair-deficient cells generally increased delayed chromosome aberrations but notably, HR-deficient 51D1 showed significantly higher chromosome aberrations than other cells (Figure 7c). Lastly, endoreduplication formation was also analyzed. DNA repair-deficient cells showed increased frequency of endoreduplication formation, especially in PARP-deficient PADR9 cells (Figure 7d).

### 2.8. Loss of BRCA2 Causing Hypersensitivity to EdU

**Figure 8:**
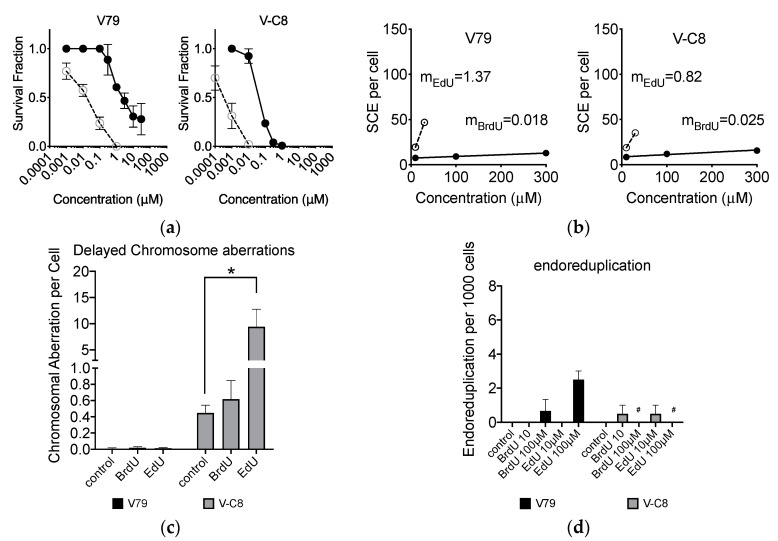
BrdU and EdU effects for Chinese hamster lung origin V79 cells and their BRCA2-deficient V-C8 cells. (**a**) Cytotoxicity with BrdU or EdU treatment; (**b**) SCE formation with BrdU or EdU treatment; (**c**) delayed effect for genomic damages after 10 μM BrdU or EdU; (**d**) endoreduplication formation after BrdU or EdU treatment. Closed circles indicate BrdU treatments and open circles indicate EdU treatments. Error bars represent the standard error of the mean of three independent experiments. One-way ANOVA, Dunnett’s Multiple Comparison Test was performed to provide *p*-values. * indicates statistically significant differences. n.s. stands for not significant (*p* > 0.05). # indicates no data available due to high toxicity.

Although CHO cells do not have isogenic BRCA2-deficient cells, Chinese hamster lung origin V79 has isogenic BRCA2-deficient V-C8 cells. Homologous recombination-deficient V-C8 cells were more sensitive to BrdU and EdU than V79 cells (Figure 8a). V-C8 showed less induction of EdU-induced SCE than V79 cells (Figure 8b). Long term effect of BrdU and EdU incorporation showed that V-C8 cells were severely sensitive to EdU for chromosome aberration formation (Figure 8c). For endoreduplication, V79 with 100 μM treatment presented 2.5 endoreduplication per 1000 cells (Figure 8d). On the other hand, V-C8 data were not available due to high toxicity at this concentration. All tested results were matched with the results obtained by CHO origin homologous recombination repair-deficient 51D1 and irs1SF cells.

### 2.9. Effect of EdU Treatment to Gamma-Rays, UVC, and Fluorescent Light

**Figure 9:**
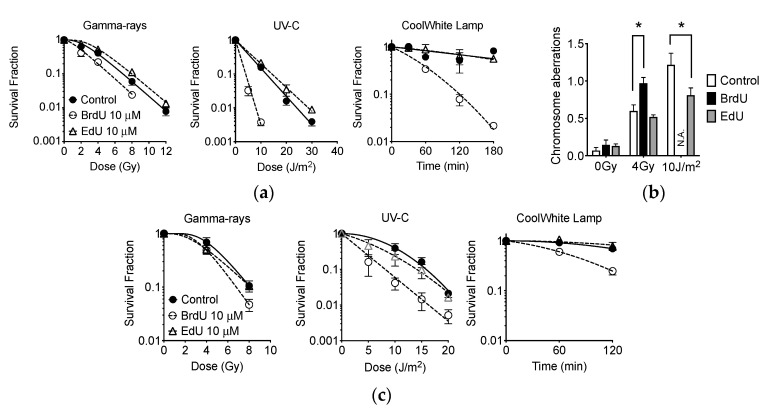
Sensitization to photon exposure with 10 μM BrdU and EdU treatment. (**a**) CHO cell survival curves with gamma-rays, UV-C, and light from fluorescent lamp. Closed circles indicate control, open circles indicate BrdU treatments, and open triangles indicate EdU treatment; (**b**) chromosome aberration analysis. Open bars indicate control. Black bars indicate BrdU treatment. Grey bars indicate EdU treatment; (**c**) A549 cell survival curves with gamma-rays, UV-C, and light from fluorescent lamp. Error bars represent the standard error of the mean of three independent experiments. One-way ANOVA, Dunnett’s Multiple Comparison Test was performed to provide *p*-values. (*) denotes significance against control within each cell group (*p* < 0.05).

CHO cells were treated with 10 μM BrdU or EdU for one cell-cycle to ensure nucleoside uptake. Cells were then irradiated in G1 phase after synchronization via mitotic shake off. Colony formation assay was then carried out to investigate potential EdU-induced photosensitization. Data from colony formation reported that, while BrdU produced an increase in sensitivity to gamma-rays, UV-C, and light from a fluorescent lamp, EdU did not (Figure 9a). The D_10_ values, the dose required to produce 10% survival, were then calculated to compare the degree of sensitization by EdU or BrdU treatment. BrdU sensitizes cells to UV-C corresponding to a decrease in D_10_ value from 12 (control) to 4.0 J/m^2^; threefold sensitization (*p* < 0.05). Surprisingly, EdU-treated cells displayed a D_10_ = 14 J/m^2^ with concomitant UV-C exposure. Thus, EdU-induced UV-C sensitization was not observed. BrdU sensitized cells to gamma-ray irradiation corresponding to a decrease in D_10_ value from 6.9 (control) to 5.3 Gy; 1.3-fold sensitization (*p* < 0.05). On the other hand, D_10_ value for EdU was calculated as 8.0 Gy. Significantly, enhanced fluorescent radiosensitization through EdU treatment was not observed. BrdU sensitizes cells to fluorescent light, with 180 min of fluorescence exposure reducing the surviving population by 98%. Contrastingly, control- and EdU-treated cells demonstrated more than 50% survival when subjected to 180 min of fluorescence exposure.

A failure of sensitization of EdU to photon exposure was confirmed with a chromosome aberration assay (Figure 9b). BrdU produced more dicentric chromosomes with 4 Gy of gamma-ray irradiation (*p* < 0.05). For BrdU-treated cells, 10 J/m^2^ of UV-C exposure was extremely toxic; thus, no metaphase chromosomes were obtained. Interestingly, EdU-treated cells showed a reduced quantity of chromatid exchanges, significantly different when compared to control cells with UV-C exposure (*p* < 0.05). Further confirmation was carried out with A549 human lung cancer cells (Figure 9c). BrdU photosensitized A549 to gamma-ray, UV-C, and fluorescent light, however, EdU did not.

## 3. Discussion

This study has revealed clearly that EdU is a strong cytotoxic Figure 1, Figure 5, Figure 6 and Figure 8 and genotoxic agent (Figure 2, Figure 3, Figure 4, Figure 7 and Figure 8). Interestingly, EdU and its derived halogenated analogs were originally used for inactivation of particular viral strains [28]. A concentration of 0.039 µM EdU induced total Herpes Simplex Virus inactivation [7]. In this report, the latter concentration (nM) induced similar cytotoxicity observed for homologous recombination-deficient cells when cultured in nucleotide/nucleoside free media (Figure 5). BrdU IC_50_ values changed from 150 μM in nucleotide/nucleoside-supplemented media to 40 μM in nucleotide/nucleoside-deficient media (Figure 1a,b). On the other hand, IC_50_ values for EdU drastically changed from nucleotide/nucleoside-supplemented media to nucleotide/nucleoside-deficient media: 75 to 0.05 μM, respectively. This differential alteration in IC_50_ values suggests that cells can tolerate BrdU incorporation into DNA, and that DNA can be reliably replicated when BrdU is conformed in the template strand. In contrast, EdU is inherently cytotoxic, and cells cannot readily use an EdU-containing strand as a template during DNA replication. Likely, an EdU-containing strand leads to replication fork stalling or collapse during DNA replication. Insofar as replicative DNA polymerases retain high-fidelity, they will not continue to extend beyond the DNA lesion (EdU-induced). Additionally, EdU contains an alkyne moiety instead of methyl group. The alkyne is covalently bonded to an azide, resulting in a triazole ring with a rigid, inflexible amide bond [29]. The triazole, we propose, has biological implications: (1) “locks-in” the nucleotide in the polymerase unable for substitution or (2) this form of nucleoside lacks 3′-oxygen atom which may cause DNA polymerase to misread the triazole-containing DNA template. Therefore, our findings suggest that when cells encounter thymidine-analog or thymidine as a template, replicative DNA polymerase may have the propensity to incorporate a nucleotide opposite thymidine-analog but remain as efficient as a nucleotide opposite thymidine.

It is necessary to note that manufacturers of EdU recommend practicing an EdU concentration of more than 10 µM in vitro. However, at such concentrations, passaging cells in media without nucleotides and nucleosides cannot tolerate continuous culture passaging (Figure 1). Additionally, the genotoxic effect of EdU persists long after EdU treatment (Figure 2 and Figure 7). Indicating that the effects may be implicated and carried through to progeny. Genomic instability and endoreduplication formation were strongly linked with EdU treatment and DNA repair capacity (Figure 2). Endoreduplication is the process by which cells replicate their genome without cytokinesis. Halogenated pyrimidine (including BrdU) induces endoreduplication [30]. In addition, a study reported a correlation between endoreduplication events and increased homologous recombination events [31]. Therefore, we suggest that EdU is implicated in the formation of replication fork stall when polymerase encounters EdU in the template strand during homologous recombination.

Our study showed that EdU induces an unavoidable amount of SCE (Figure 4). Our data report that the number of 100 SCEs per cell was nearly similar in degree to cells observed from Bloom syndrome and XRCC1-mutated CHO EM9 cells [32,33]. Cells subject to EdU distinctly demonstrate that, at high concentrations of EdU (>10 µM), uptake induces replication stress and activates the recombination process as observed in gamma-H2AX and Rad51 foci formation (Figure 3). Although SCE is considered a highly error-free event, elevated HPRT mutation frequency was observed with EdU treatment in this study (Figure 2). It may be explained by an incomplete SCE reparation due to an incorporated EdU-induced replication fork collapse or stress (Figure 7a,b). It is worth noting that two HR-deficient mutant cell lines 51D1 and irs1SF showed significantly elevated SCE induction with high concentration of EdU treatment but not with BrdU (Figure 4). This suggests that SCEs induced through EdU may be Rad51 paralog/Rad51 independent events. This was supported with V-C8, V79 origin BRCA2 mutants (Figure 8). Additionally, our reported high SCE frequency may explain the high sensitivity to EdU in HR-deficient cells (Figure 5 and Figure 6). Additionally, since we observed an increase in SCE formation and chromosomal aberrations in second post-EdU-labeled metaphases in 51D1 cells among tested DNA repair-deficient mutants (Figure 7a,b), we suggest that EdU did not inhibit DNA synthesis. Rather, it is more likely that DNA replication fork collapse occurs when the EdU-incorporated DNA strand was used as the template rather than BrdU [19]. Moreover, homologous recombination repair manages those replication fork collapses (Figure 3b). In the absence of HR repair and presence of EdU, chromosomal aberrations are highly probable even observed in delayed (Figure 7b,c).

Ultimately, EdU-induced DNA damage or stress is repaired by the HR pathway (Figure 3). However, when HR is not available or defective, it induces chromosomal aberrations (Figure 7) and is notably toxic. Homologous recombination repair with functional Rad51 alleviates DNA damage response induced by EdU. The severe toxic effects of EdU may be due to the actual structural differences compared to BrdU. The Van der Walls radius of the Br atoms is 19–19.7 nm [34]. This is close to that of a CH_3_ group (20 nm). The volume of the ethynyl group (28.8 Å^3^) is similar that of Br (24.4 Å^3^), but, their shapes are chemically different. The ethynyl substituent, -C≡CH, retains 2 carbon atoms and a cylindrical shape [35]. In addition, it is well known throughout organic chemistry that triple bonds are highly reactive in nature. Therefore, the triple bond in the ethynyl moiety may be reacting in an adverse or unwanted manner. Although the structure of thymidine analogs are similar, these various minute changes at the subcellular metric are extremely important to the specificity of DNA polymerases. However, incorporation of EdU may not be a suitable template for DNA synthesis. As a result, EdU treatment results in cellular cytotoxicity and genotoxicity.

When monitoring cellular kinetics, it is clearly advantageous to utilize EdU, compared to BrdU: lower cellular toxicity is achieved when cells are exposed to light from fluorescent sources (Figure 9). This means that one can manipulate or transfer cells incubated in EdU and in the presence of fluorescent light with a higher degree of confidence. Interestingly, EdU did not achieve cellular sensitization to gamma-rays, UV-C, or light from a fluorescent lamp (Figure 9). BrdU labeling provoked unexpected phototoxicity in an in vitro or in vivo environment when exposed to illuminated rooms or light from microscopes. Therefore, researchers may find it beneficial to use EdU, easing cell culture handling and long-term live cell microscopic analysis; ultimately avoiding unnecessary phototoxicity. It was unclear why EdU incorporation reduced UV-C-induced chromosome aberrations and slightly increased survival of CHO cells after UV-C exposure (Figure 9b).

## 4. Materials and Methods

### 4.1. Cell Cultures

Chinese Hamster Ovary (CHO) wild type CHO10B2, DNA repair-deficient V3 [36], xrs5 [37], XR1 [32] and PADR9 [33], Chinese hamster lung origin V79 and DNA repair-deficient V-C8 [38], and A549 human lung cancer cells were kindly supplied by Dr. Joel Bedford (Colorado State University, Fort Collins, CO, USA). DNA repair-deficient CHO cells 51D1 [39], KO40 [40], irs1SF [41] were kindly supplied by Dr. Larry Thompson (Lawrence Livermore National Laboratory, Livermore, CA, USA). V3, xrs5, XR1 are non-homologous end joining (NHEJ) repair-deficient cells and 51D1, irs1SF, and V-C8 are homologous recombination (HR) repair-deficient cells. KO40 has Fanconi Anemia pathway deficiency. PADR9 has poly (ADP-ribose) formation deficiency. Cells were cultured in Eagle’s minimal essential medium with alpha modification (Gibco, Indianapolis, IN, USA), supplemented with 10% heat-inactivated fetal bovine serum (FBS; Millipore-Sigma, St. Louis, MO, USA), 1% penicillin, streptomycin, and fungizone (Gibco, Indianapolis, IN, USA), and maintained at 37 °C in a 5% CO_2_ incubator. Cell cultures were subcultured when necessary.

### 4.2. Cell Doubling Time Analysis

In total, 10,000 cells were plated in either the absence or presence of DMSO (control), followed by the addition of either BrdU or EdU at varying concentrations. The cell number was quantified by a Coulter Counter Z1 (Beckman Coulter, Brea, CA, USA). Cell number was then counted every 12 h for four days. Cell doubling time was calculated via GraphPad Prism 6 (GraphPad, La Jolla, CA, USA) with regression analysis [42].

### 4.3. Colony Formation Assay

In order to investigate the cytotoxicity of EdU and BrdU, a colony formation assay was carried out with alphaMEM either with or without nucleotide and nucleoside supplemented media. Cells were then treated with various concentrations of BrdU or EdU. Cells were fixed with 100% ethanol and stained with crystal violet solution 8 days after the incubation period. Colonies were observed under a light microscope and colonies containing more than 50 cells were counted as survivors. MEM media, which does not contain nucleotide and nucleoside, was used to investigate EdU cytotoxicity for DNA repair-deficient cells. Cell survival assay was carried out a minimum of 3 times, independently. Survival curves were fitted with a sigmoidal model by GraphPad Prism 6 software.

### 4.4. DNA Damage Repair Responses Assay

BrdU-and EdU-induced DNA damage repair responses were investigated via immunocytochemistry. Cells were treated with 10 μM of BrdU or EdU for 24 h. Then cells were fixed in 4% paraformaldehyde and permeabilized by 0.5% Triton X-100 and 0.1% SDS. After blocking in 10% goat serum, mouse monoclonal antibody for anti-gamma-H2AX (Millipore-Sigma), rabbit polyclonal antibody for Rad51 (Santa Cruz Biotechnology, Santa Cruz, CA, USA), and rabbit polyclonal antibody for FancD2 (Novus Biological, Centennial, CO, USA) was used as previously described [43]. Secondary antibody was Alexa Fluor 488 or Alexa Fluor 594 conjugated antibody. Cells were mounted in a solution of 2 μg/mL DPI containing slow-fade (Invitrogen, Carlsbad, CA, USA). Fluorescent images were captured using a Zeiss Axioskop fluorescent microscope (Zeiss, Oberkochen, Germany) equipped with Q-imaging Aqua Camera (Q-imaging, Montreal, QC, Canada) with Q-capture pro software. Three independent experiments were performed. Manual counting was performed for 50 cells to obtain the average number of foci per cell. Signal intensity was obtained with Q-capture pro software.

### 4.5. Chromosomal Aberration Analysis for Long Term Treatment

BrdU- and EdU-induced cytogenetic damage was investigated in metaphase-spread chromosomes. Cells were treated with 100 μM of BrdU or EdU for 24 h. After changing and replenishing media without BrdU or EdU, cells were cultured for an additional 24 h. In the last 6 h, Colcemid was added to induce metaphase arrest. Cells were harvested during metaphase, trypsinized and then suspended in 4 mL of 75 mM KCl solution warmed to 37 °C and placed in a 37 °C water bath for 20 min. A Carnoy’s solution (3:1 methanol to acetic acid) was added to the samples according to the standard protocol [44]. Fixed cells were dropped onto slides and allowed to dry at room temperature. Slides were stained with 5% Giemsa solution in Gurr buffer. Chromosomal aberration analysis was carried out under a Zeiss Axioskop microscope. Fifty metaphase cells were analyzed for chromosomal aberration analysis. Images were captured by a Diagnostic Instrument SPOT camera and software. Overall, 1000 metaphase cells were analyzed for endoreduplication formation analysis [30,45]. Three independent experiments were performed.

### 4.6. Chromosomal Aberration Analysis for Short Term Treatment

In order to identify the critical treatment time of BrdU or EdU to induce chromosome aberrations, cells were treated with BrdU or EdU at different times before metaphase harvest. Cells were treated at the same cell cycle with BrdU or EdU, or treated with BrdU or EdU for 1 cell cycle duration, then cultured in a media without BrdU or EdU for an additional cell cycle. Metaphase chromosome aberration analysis was performed using the same protocol as the long-term treatment experiment (Section 4.5).

### 4.7. Hypoxanthine Phosphorybosyl Transferase (HPRT) Mutation Analysis

Prior to use in the HPRT mutation assay, CHO wild type cells were grown in MEM media with HAT supplement (2 × 10^−4^ M hypoxanthine, 2 × 10^−7^ M aminopterin, and 1.75 × 10^−5^ M thymidine) for two days in order to reduce the level of spontaneous HPRT mutations. Then, the cells were recovered in regular MEM media for two days. After incubating cells, 1 μM BrdU or 1 μM EdU was added for 24 h. After drug treatment, single-cell suspensions were diluted and grown in a nonselective medium for a period of time sufficient to allow phenotypic expression prior to plating for determination of mutant frequency. Phenotypic expression time is approximately 7 days for HPRT mutation. Then, 3 × 10^5^ cells were plated in P60 cell culture dishes with a selective agent, 6-thioguanine (6-TG, 5 μg/mL). Cells from corresponding dishes were also plated at a low density (300 cells/mL) in the absence of the selective agent to determine plating efficiency. All cell cultures were incubated for 10 days, permitting a sufficient growth period prior to scoring the colonies. Each HPRT mutation experiment was carried out a minimum of 3 times to ensure reproducibility [46,47].

### 4.8. Sister Chromatid Exchange

CHO cells were synchronized into the G1 phase using a mitotic shake-off procedure [9,48]. Synchronized mitotic cells were subcultured in T25 flasks and incubated for two hours at 37 °C. Cells were treated with 10 μM of BrdU for two cell cycles; 0.2 μg/mL of colcemid (Gibco, Indianapolis, IN, USA) was added to cell medium and allowed to incubate for an additional 6 h. Cells were harvested during metaphase, trypsinized and then resuspended in 4 mL of 75 mM KCl solution warmed to 37 °C and placed in a 37 °C water bath for 20 min. A Carnoy’s solution (3:1 methanol to acetic acid) was added to the samples according to the standard protocol [43]. Fixed cells were dropped onto slides and allowed to dry at room temperature. Differential staining of metaphase chromosomes was completed using the fluorescence plus Giemsa technique by treating chromosomes with Hoechst 33258 followed by UV-A exposure [49].

SCE was prepared with EdU as followed: EdU was added to exponentially growing cell culture for 12 h. Mitotic shake off method was carried out to obtain a G1 population with 1 cycle of EdU treatment. Synchronized cells were cultured in fresh media without EdU and first post-metaphase was harvested with Colcemid treatment. Metaphase chromosome spread was prepared via the BrdU-SCE method above. EdU staining was carried out by way of Click reaction with Alexa 488-azide. DNA was counter-stained by DAPI. Differentially stained metaphase chromosome images were taken using a Zeiss Axioplan microscope (Zeiss, Oberkochen, Germany) equipped with Q-imaging Aqua CCD camera and Q-capture Pro software (QImaging, Surrey, BC, Canada). Fifty metaphase cells were scored for each treatment concentration. Data presented are the mean of SCE frequency per cell.

### 4.9. Sensitization Effects of BrdU and EdU to UV-C, Gamma-Rays, and Light from Fluorescent Lamp

Phillip’s germicidal UV-C lamps were used as a UV-C source with a fluence of 1 W/m^2^. Dosimetry was carried out using a UVP UVX dosimeter with a UV-C probe (UVP, Upland, CA, USA) [50]. Cell culture dishes were rotated at 6 rpm during UV-C exposure to ensure even exposure to all areas of the dish. Gamma-ray irradiation was carried out using a J.L. Shepherd Model Mark I-68 222 TBq (6000 Ci) 137Cs irradiator. The dose rate was 2.5 Gy/min. Fluorescent light exposure was carried out with a Coolwhite lamp (Phillips). Exposure to fluorescent light was carried out 20 cm below light source with an illuminance of 3000 lux. Dosimetry was carried out with an Urceti Light meter (Shenzhenshi Qijiajia Dianzishangwu Co Ltd., Guangdong, China).

Exponentially growing cells were cultured with 10 μM of BrdU or EdU for 12 h. Mitotic shake off method was carried out to obtain G1 phase cells with BrdU or EdU treatment [23]. Following appropriate photon exposure and trypsinization, the cells were plated to form colonies. The cultures were then fixed with absolute ethanol and stained with 0.1% crystal violet. Lastly, colonies containing more than 50 cells were scored as survivors. Sensitization was quantitatively evaluated through calculating the D_10_ values of control vs. Brd- or EdU-treated cells.

Cytotoxicity was confirmed through first post-irradiated metaphase chromosome analysis. Cells were treated with Colcemid and metaphase cells were treated with 75 mM KCl solution and fixed in 3:1 methanol: acetic acid solution. Exchange type chromosome aberrations (dicentrics and chromatid-type aberrations) were analyzed under a Zeiss Axioskop microscope. At least 50 metaphase cells were analyzed.

### 4.10. Statistics

All studies were conducted as independent experiments for a minimum of three times for each endpoint, and error bars indicate standard errors of the means. Statistical significance was assessed by implementing a One-way ANOVA, using Prism 6 software (GraphPad Software, San Diego, CA, USA). For all data, *p* < 0.05 indicate statistical significance.

## 5. Conclusions

In conclusion, EdU is significantly much more toxic compared to BrdU in terms of cytotoxicity and mutagenic potential. In particular, researchers using any DNA repair-deficient cells should proceed with caution and agency to avoid these potential cellular implications. Cell culture media selection is also very important for EdU labeling. Additionally, media without thymidine supplementation such as, MEM, DMEM, and RPMI should be used cautiously. Lastly, researchers should pay maximal attention to EdU concentrations (10–300 µM) to avoid unexpected or unwanted toxicity.

## Figures and Tables

**Table 1:** IC_50_ values of BrdU and EdU for DNA repair-deficient cells.

Cell Line	BrdU (μM)	EdU (μM)	Ratio BrdU/EdU	Affected Pathways
CHO	15	0.088	170	n.a.
V3	0.54	0.025	21	NHEJ
xrs5	0.63	0.023	27	NHEJ
XR-1	0.56	0.022	25	NHEJ
irs1SF	0.30	0.0027	111	HR
51D1	0.54	0.0021	257	HR
KO40	0.44	0.011	40	Fanconi Anemia
PADR9	0.38	0.010	38	poly(ADP-ribosylation)
